# Interplay between fungicides and parasites: Tebuconazole, but not copper, suppresses infection in a *Daphnia-Metschnikowia* experimental model

**DOI:** 10.1371/journal.pone.0172589

**Published:** 2017-02-23

**Authors:** Ana P. Cuco, Nelson Abrantes, Fernando Gonçalves, Justyna Wolinska, Bruno B. Castro

**Affiliations:** 1 Department of Biology, University of Aveiro, Aveiro, Portugal; 2 Centre for Environmental and Marine Studies (CESAM), University of Aveiro, Aveiro, Portugal; 3 Department of Environment and Planning, University of Aveiro, Aveiro, Portugal; 4 Department of Ecosystem Research, Leibniz-Institute of Freshwater Ecology and Inland Fisheries (IGB), Berlin, Germany; 5 Department of Biology, Chemistry, Pharmacy, Institute of Biology, Freie Universität Berlin, Berlin, Germany; 6 Centre of Molecular and Environmental Biology (CBMA), Department of Biology, University of Minho, Braga, Portugal; Universiteit Gent, BELGIUM

## Abstract

Natural populations are commonly exposed to complex stress scenarios, including anthropogenic contamination and their biological enemies (e.g., parasites). The study of the pollutant-parasite interplay is especially important, given the need for adequate regulations to promote improved ecosystem protection. In this study, a host-parasite model system (*Daphnia* spp. and the microparasitic yeast *Metschnikowia bicuspidata*) was used to explore the reciprocal effects of contamination by common agrochemical fungicides (copper sulphate and tebuconazole) and parasite challenge. We conducted 21-day life history experiments with two host clones exposed to copper (0.00, 25.0, 28.8 and 33.1 μg L^-1^) or tebuconazole (0.00, 154, 192 and 240 μg L^-1^), in the absence or presence of the parasite. For each contaminant, the experimental design consisted of 2 *Daphnia* clones × 4 contaminant concentrations × 2 parasite treatments × 20 replicates = 320 experimental units. Copper and tebuconazole decreased *Daphnia* survival or reproduction, respectively, whilst the parasite strongly reduced host survival. Most importantly, while copper and parasite effects were mostly independent, tebuconazole suppressed infection. In a follow-up experiment, we tested the effect of a lower range of tebuconazole concentrations (0.00, 6.25, 12.5, 25.0, 50.0 and 100 μg L^-1^) crossed with increasing parasite challenge (2 *Daphnia* clones × 6 contaminant concentrations × 2 parasite levels × 20 replicates = 480 experimental units). Suppression of infection was confirmed at environmentally relevant concentrations (> 6.25 μg L^-1^), irrespective of the numbers of parasite challenge. The ecological consequences of such a suppression of infection include interferences in host population dynamics and diversity, as well as community structure and energy flow across the food web, which could upscale to ecosystem level given the important role of parasites.

## Introduction

A multiple stressor framework is required to understand the effects of environmental change under an ecologically relevant perspective [1–3]. Given the need for adequate regulations for ecosystem protection, it is especially important to consider interaction scenarios involving anthropogenic contaminants. However, standard ecotoxicity tests determine the effects of single contaminants only, and may thus under- or overestimate hazards if interactive scenarios with other stress factors are not considered [2,4]. Contaminants that have no impact on organisms individually may be harmful when other stressors are present [4,5]. For instance, the sensitivity of organisms may be altered when they are simultaneously exposed to environmental changes [2], such as temperature increase [3,6,7] or reduced food availability [8,9]. Sensitivity to contaminants may also be altered by specific biotic agents, such as parasites or predators [2,10]. Within a risk assessment framework, it is therefore desirable to focus on multiple stress scenarios, because they can provide a more accurate and realistic prediction than individual stressor effects [4,9].

The impacts of agricultural activity are particularly relevant to the need for a multiple stressor framework to adequately assess the risks to non-target organisms and to protect ecosystem services. Extensive agricultural exploitation poses major environmental concerns due to the release of organic and inorganic contaminants into the environment [11,12]. Fungicides, which often account for the majority of pesticide application, are extensively used to fight fungal parasites, particularly in vineyards [12,13]. Copper-based and azole fungicides are some of the most commonly used compounds [14]; copper is a general biocide that can cause growth inhibition and interfere with several cellular processes [15], and azoles are a family of highly effective systemic fungicides that act by inhibiting ergosterol biosynthesis [14,16]. Their broad use and persistence results in their frequent detection in aquatic systems [12,14,17]. Fungicides reach superficial waters via runoff events, leaching or spray-drift, and may cause negative effects in natural populations and ecosystem processes, such as leaf litter breakdown [13]. The ecological effects of copper and azole compounds must therefore be assessed when monitoring surface and groundwater quality of aquatic systems near agriculture activity [11].

Pesticides–and fungicides in particular–are designed to eliminate nuisance species (including parasites) that compromise agricultural yield. However, disease is an important natural stressor, and it is omnipresent in all types of ecosystems [18]. Parasitism has an important role in modulating host population dynamics and community structure, via parasite-induced host mortality or reduced fecundity, as well as frequency-dependent selection [19]. Furthermore, parasites are important drivers of genetic diversity in host populations, as demonstrated in experimental and field studies [20,21]. Host-parasite relationships are strongly dependent on environmental context [22], and their outcome can be altered by the presence of natural and anthropogenic stressors, including pesticides [2,23]. Interactive scenarios between parasites and pesticide exposure have been shown to be mostly synergistic, causing exacerbated impacts on the host´s sensitivity to the pollutant [5,24] or on their susceptibility to infection [25,26]. However, this may not always be the case [27,28]. Contaminants may either increase or decrease parasite fitness and transmission rates, the latter happening if parasites are more susceptible to pollutants than their hosts, or if pollutants negatively affect intermediate hosts or vectors (see rationale and examples in [29]).

Bearing in mind the putative reciprocal effect of disease and pollution, we investigated the interaction between parasitism and fungicides using a host (*Daphnia* spp.) × parasite (*Metschnikowia bicuspidata*) model system. Life history assays with this model system allow us to discriminate the relative contribution of contaminant exposure and parasite challenge to the outcome of the host-parasite relationship. Tebuconazole and copper sulphate were selected as contaminants due to their common use as fungicides in agriculture and frequent presence in superficial and runoff waters [12,30,31]. We performed two experiments: first, we tested the effect of each contaminant in the presence or absence of the parasite; second, we tested the effect of increasing parasite challenge (single vs. double challenge) on the response of the host-parasite system to the contaminant. Our null hypothesis (H_0_) assumed no contaminant × parasite interaction (i.e., independent effects). However, we predict the existence of interactive effects, via (H_1_) contaminant-induced reduction of host fitness or immunity, leading to increased parasite infectivity or virulence (we foresee this scenario for copper, given its generalist mode of action and high toxicity to *Daphnia*); or (H_2_) contaminant-induced reduction of parasite fitness leading to decreased parasite infectivity or virulence (we foresee this for tebuconazole, given its specific antifungal mode of action). In the presence of such interactions, we expect that an increase in the number of parasite challenges, as applied in the second (follow-up) experiment, would exacerbate the parasite effects (if H_1_ true) or offset the anti-parasitic effect of the contaminants (if H_2_ true).

## Methods

### Host-parasite system: Origin and maintenance

The water flea *Daphnia* spp. is a model organism in aquatic ecology, ecotoxicology and evolution, because of its life cycle characteristics convenient for experimental testing (short life cycle and cyclic parthenogenetic reproduction) and central position in aquatic food webs [32,33]. Two genotypes belonging to the *Daphnia longispina* species complex (clone AMM_12—*D*. *galeata x longispina* hybrid and clone AMM_47—*D*. *galeata*; hereafter designated as clone 12 and clone 47, respectively) were selected as model hosts, and maintained as single-cohort group cultures in the laboratory. These *Daphnia* genotypes were isolated from Lake Ammersee (Germany), and they are part of a collection of *Daphnia* clones that have been used in other studies [23,34]. The use of more than one genotype/taxon increases ecological relevance, but the use of numerous clones is often not logistically feasible. The use of two distinct taxonomical entities (clones 12 and 47) represents a compromise that allows a more robust assessment of the observed patterns. *Daphnia* cultures were reared in moderately hard reconstituted water (123 mg L^-1^ MgSO_4_·7H_2_O, 96 mg L^-1^ NaHCO_3_, 60 mg L^-1^ CaSO_4_·2H_2_O, 4 mg L^-1^ KCl) supplemented with a standard organic additive (algal extract) and vitamins (for a more detailed description, see [8] and [32]). Group cultures consisted of 30–40 individuals in 800 ml of culture medium, which were kept in a controlled temperature room (at 20 ± 1°C) with a 16h^L^:8h^D^ photoperiod. Medium was renewed three times per week and, at each medium renewal, daphniids were fed with *Raphidocelis subcapitata* at a ration of 1.5 x 10^5^ cells mL^-1^. Several synchronized group cultures per clone were used to warrant a sufficient number of juveniles for the experiments. To assure homogeneous quality and standardization, neonates (less than 24 h old) born between the 3^rd^ and 5^th^ broods were used to renew the cultures or to carry out the experiments.

The microparasite *M*. *bicuspidata* is a yeast that grows inside *Daphnia*´s body cavity and produces needle-like ascospores (S1 Fig) that are released from dead infected hosts, and then grazed upon by healthy *Daphnia* (horizontal transmission); in this process, the parasite reduces the host’s fecundity and lifespan [33]. A logistical advantage of this model is that *M*. *bicuspidata* can be cultured inside its host, and spore suspensions can be obtained from infected *Daphnia*, allowing control of parasite transmission and spore load in infection experiments [34–36]. The endoparasitic yeast *M*. *bicuspidata* was also isolated from Lake Ammersee and was maintained in an infected *Daphnia magna* culture. Uninfected *D*. *magna* were added to infected cultures every other week to guarantee a continuous supply of hosts and to ensure parasite transmission.

### Test chemicals

Tebuconazole (Tebuconazol PESTANAL^®^, CAS nr. 107534-96-3) [(RS)-1-p-chlorophenyl)-4,4-dimethyl-3-(1H-1,2,4-triazol-1-ylmethyl)pentan-3-ol] and copper sulphate pentahydrate (CuSO_4_·5H_2_O, CAS nr. 7758-99-8) were obtained from Sigma Aldrich (Munich, Germany) and Merck (Darmstadt, Germany), respectively. Concentrated stock solutions were prepared in distilled water (for copper sulphate) or ethanol (for dissolving tebuconazole) and then diluted in reconstituted water to obtain final test solutions: 0.00, 154, 192 and 240 μg L^-1^ for tebuconazole and 0.00, 25.0, 28.8 and 33.1 μg L^-1^ for copper sulphate (expressed as μg of free Cu^2+^ ion per litre). Aqueous solutions of tebuconazole and copper are not easily degraded; tebuconazole has a half-life of 28–43 days whilst copper is considered a stable aqueous solution [37,38]. Our goal was to set a geometric series of non-lethal concentrations causing reproductive impairment in *Daphnia* spp., based on a previous study [3]. For tebuconazole exposure, ethanol was also added in control replicates (0.00 μg L^-1^) at an equal volume to the one used in the tebuconazole concentrations (0.1 ml L^-1^).

In order to check the validity of the nominal concentrations tested, aliquots of the lowest and the highest tested concentrations of all experiments were collected at two random timings and sent to an independent analytical laboratory (certified according to ISO/IEC 17025:2005). Quantification of tebuconazole was performed by liquid chromatography mass spectrometry (LC-MS/MS), according to an internal method adapted from ISO 11369:1997. Copper (Cu) was analysed by inductively coupled plasma mass spectrometry (ICP-MS) after digestion with HNO_3_, according to ISO 17294–2:2003. Analytical data confirmed the validity of nominal concentrations in all experiments (see Experimental design and Follow-up experiment). Measured concentrations slightly overestimated nominal concentrations: 10–16% for copper and 1–18% for tebuconazole (S1 Table). Given this data, we assumed a good correspondence between nominal and measured concentrations.

### Experimental design

Life history assays were conducted for 21 d with both *Daphnia* clones exposed to a range of tebuconazole or copper sulphate concentrations (see above) and the presence (“parasite”) or absence (“no-parasite”) of the parasite *M*. *bicuspidata*, in a fully crossed design. For logistical reasons, experiments were carried out separately for each contaminant. Thus, experimental design for each contaminant consisted of 2 *Daphnia* clones x 4 contaminant concentrations x 2 parasite treatments x 20 replicates = 320 experimental units. Each experiment was initiated by individually placing neonates in glass vessels with 50 mL of corresponding test solution. Assays were carried out under the same light and temperature conditions as the cultures. The medium was renewed every three days and daphniids were fed daily (with a *R*. *subcapitata* ration of 1.5 x 10^5^ cells mL^-1^). With the necessary adaptations, test procedures followed OECD [39] recommendations. Individual *Daphnia* were infected on day 5 with an *M*. *bicuspidata* spore suspension (parasite treatment) or a placebo (no-parasite treatment), following the methodology described below.

Survival and reproductive parameters were recorded daily for each individual. Reproductive parameters included age at first reproduction (AFR), fecundity at day 14 (cumulative number of living offspring released per surviving female at day 14) and reproductive output at day 21 (cumulative number of living offspring released per female). Fecundity was estimated at day 14, because its calculation at day 21 was impacted by parasite-mediated mortality, which was not yet the case on day 14. Reproductive output (measured at day 21) considers the combined effects of stressors on both survivorship and fecundity [3,39]. For the calculation of reproductive output, we excluded replicates in which test organisms died before day 4, as this could be the result of accidental or natural early-age mortality. Individuals were visually inspected, under an Olympus CKX41 inverted microscope, for confirmation of infection (presence of spores inside body cavity–S1 Fig) at time of death or at the end of the assay (day 21). Infection percentages were calculated in each treatment. Survival and reproduction data at the end of the experiment (day 21) were used to determine the per capita intrinsic rate of population increase (*r*) from the Euler-Lotka equation:

$$1=\overset{n}{\underset{x=0}{\sum }}e^{-rx}l_{x}m_{x},$$

where *r* is the rate of population increase (day^-1^), *x* is the age class in days, *l*_*x*_ is the probability of surviving to age *x*, and *m*_*x*_ is the fecundity at age *x*. Replicate pseudo values for *r* were generated using the jack-knifing technique described by Meyer et al. [40].

### Infection procedure

Infected *D*. *magna* were crushed in distilled water to obtain a homogenized *M*. *bicuspidata* spore suspension, and spore concentration was determined using a Neubauer improved counting chamber. A placebo was obtained by crushing the same number of uninfected *D*. *magna* in distilled water; this allows the possible effects of bacteria or alarm cues from the crushed conspecifics to be controlled [35].

Parasite challenge was carried out between the 5^th^ and 7^th^ day of the experiments. On the first day of infection (day 5), test solution volume from all experimental units was reduced from 50 mL to 15 mL and organisms were not fed to ensure higher filtration rates and, thus, a higher spore encounter rate [36]. Experimental units from parasite treatment were inoculated with a spore suspension aliquot (to obtain a final spore density of 700 spores mL^-1^ [35]) while experimental units from no-parasite treatment were given an equivalent placebo aliquot. On the following days, test solution volume was progressively increased to 30 mL (day 6) and 45 mL (day 7), by adding fresh medium. On day 6, daily feeding was resumed (1.5 x 10^5^ algal cells mL^-1^) and, from day 8 onwards, the initial conditions were restored (50 mL of test solution, daily feeding, and medium renewal every three days).

### Follow-up experiment

An additional life history assay was carried out in order to further explore the effects of tebuconazole on the *Daphnia*-*M*. *bicuspidata* model system. This experiment was not carried out for copper, because no toxicant × parasite interaction was observed (see Results). Here, we tested the effect of lower tebuconazole concentrations (0.00, 6.25, 12.5, 25.0, 50.0 and 100.0 μg L^-1^) and two levels of parasite challenge (“single challenge” versus “double challenge”). In the single challenge treatment, experimental units were inoculated as previously; in the double challenge treatment, inoculation was performed twice–at day 3 and 5 –thus simulating a higher spore load in total [34]. The experiment was conducted in a fully crossed design consisting of 2 *Daphnia* clones x 6 tebuconazole concentrations x 2 parasite levels x 20 replicates = 480 experimental units. All experimental procedures were similar to the previous assay, except for the parasite infection procedure. Also, we reduced the volume of test solutions, relative to the previous experiments, as a refinement step to minimize the amount of tebuconazole and spores required, as well as producing less toxicant waste. Test vessels were filled with 10 mL of corresponding test solution on day 0, and test solution volumes were progressively increased to 15 mL (day 6) and 25 mL (day 7). The experiment was terminated on day 21.

### Statistical analysis

Two-way analyses of variance (ANOVA) were performed separately for each contaminant to assess the effects of parasite presence or number of parasite challenges (first and second experiment, respectively) and contaminant concentration (fixed factors) on life history parameters of *Daphnia*. Because we were expecting differences in the sensitivity of the tested clones to parasite and contaminant (see [3]), tests were conducted separately per clone. Hence, a necessary adjustment in the significance level was performed (α = 0.025) using the Dunn-Sidak procedure. Prior to analyses, plots of residuals were analysed to assess deviations from normality and homogeneity of variances; although minor deviations were sometimes found, we assumed ANOVA to be sufficiently robust to deal with them. However, AFR was ln-transformed to comply with more pronounced heterogeneous variances. All statistical analyses were conducted using R software, version 3.1.2 [41].

## Results

**Fig 1:**
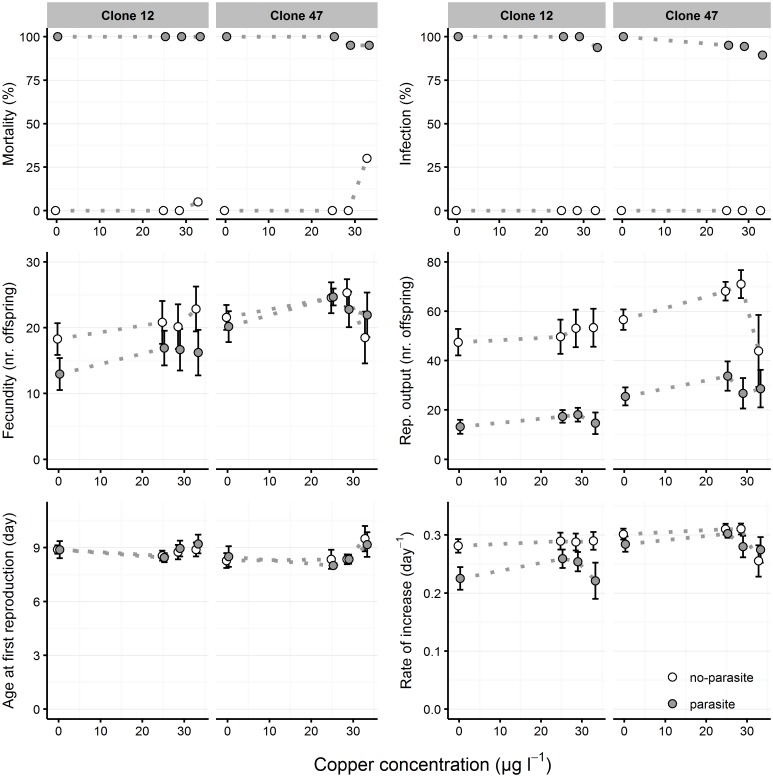
Life history parameters of *Daphnia* clones exposed to copper and parasite challenge. Two *Daphnia* clones (Clone 12 and Clone 47) were exposed to increasing copper concentrations in the absence (”no-parasite”) and presence (”parasite”) of spores of the microparasitic yeast *M*. *bicuspidata*. Host mortality and parasite infectivity are shown as the proportion of dead or infected hosts, respectively, at the end of the experiment (day 21). Fecundity (at day 14), reproductive output (at day 21), age at first reproduction and intrinsic rate of increase are shown as mean (circle) and respective 95% confidence intervals (error bars), based on 20 experimental units per treatment. Dotted lines between data points are used to facilitate visualization of the concentration-dependent response across parasite treatments.

**Fig 2:**
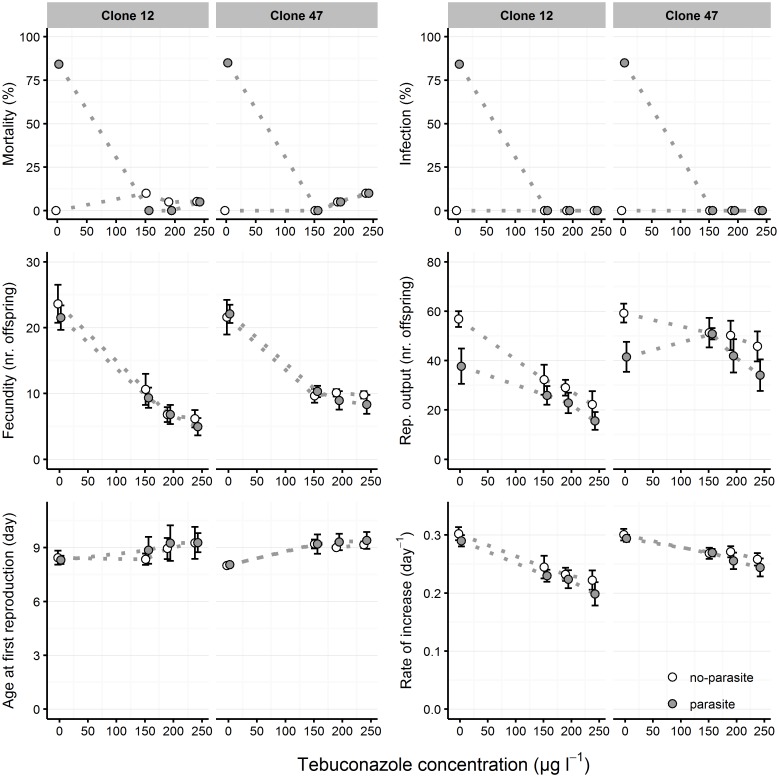
Life history parameters of *Daphnia* clones exposed to tebuconazole and parasite challenge. Two *Daphnia* clones (Clone 12 and Clone 47) were exposed to increasing tebuconazole concentrations in the absence (”no-parasite”) and presence (”parasite”) of spores of the microparasitic yeast *M*. *bicuspidata*. Host mortality and parasite infectivity are shown as the proportion of dead or infected hosts, respectively, at the end of the experiment (day 21). Fecundity (at day 14), reproductive output (at day 21), age at first reproduction and intrinsic rate of increase are shown as mean (circle) and respective 95% confidence intervals (error bars), based on 20 experimental units per treatment. Dotted lines between data points are used to facilitate visualization of the concentration-dependent response across parasite treatments.

Infection rates in the controls (i.e. no contaminant) were 85–100% for both clones, demonstrating a successful infection procedure; no individuals became infected in the no-parasite treatment. High mortality levels were observed in parasite treatment, from day 14 onwards, concordant with high levels of infection (Figs 1 and 2). Infection with *M*. *bicuspidata* was mostly independent of copper exposure and infection rates were consistently high in all concentrations (> 89.5%), for both clones (Fig 1). Conversely, infection was suppressed by tebuconazole exposure from 153.6 μg L^-1^ upwards (Fig 2).

### Copper x parasite

Parasite presence caused ca. 100% host mortality irrespective of copper concentration. In the absence of the parasite, copper caused slight mortality in the highest concentration (Fig 1). The effects of copper and parasite presence were mostly independent (see non-significant interaction terms in Table 1).

**Table 1:** Two-way ANOVA summary table of the effects of contaminant concentration and parasite challenge.

		Copper		Tebuconazole	
	Source	Clone 12	Clone 47	Clone 12	Clone 47
**ln AFR**	Concentration	F_(3, 146)_ = 3.2; ***P* = 0.003**	F_(3, 145)_ = 10; ***P* < 0.001**	F_(3, 147)_ = 4.1; ***P* = 0.008**	F_(3, 152)_ = 40; ***P* < 0.001**
	Parasite presence	F_(1, 146)_ = 0.47; *P* = 0.494	F_(1, 145)_ = 0.52; *P* = 0.472	F_(1, 147)_ = 0.66; *P* = 0.419	F_(1, 152)_ = 1.6; *P* = 0.203
	Conc. x Parasite	F_(3, 146)_ = 0.57; *P* = 0.634	F_(3, 145)_ = 0.78; *P* = 0.505	F_(3, 147)_ = 0.33; *P* = 0.800	F_(3, 152)_ = 0.56; *P* = 0.641
**Fecundity at day 14**	Concentration	F_(3, 147)_ = 3.2; *P* = 0.026	F_(3, 142)_ = 6.7; ***P* < 0.001**	F_(3, 148)_ = 166; ***P* < 0.001**	F_(3, 149)_ = 197; ***P* < 0.001**
	Parasite presence	F_(1, 147)_ = 21; ***P* < 0.001**	F_(1, 142)_ = 0.085; *P* = 0.711	F_(1, 148)_ = 3.5; *P* = 0.062	F_(1, 149)_ = 0.72; *P* = 0.397
	Conc. x Parasite	F_(3, 147)_ = 0.46; *P* = 0.711	F_(3, 142)_ = 2.2; *P* = 0.089	F_(3, 148)_ = 0.54; *P* = 0.655	F_(3, 149)_ = 1.6; *P* = 0.188
**Reproductive output**	Concentration	F_(3, 153)_ = 1.9; *P* = 0.136	F_(3, 154)_ = 8.4; ***P* < 0.001**	F_(3, 151)_ = 50; ***P* < 0.001**	F_(3, 154)_ = 6.5; ***P* < 0.001**
	Parasite presence	F_(1, 153)_ = 354; ***P* < 0.001**	F_(1, 154)_ = 159; ***P* < 0.001**	F_(1, 151)_ = 33; ***P* < 0.001**	F_(1, 154)_ = 23; ***P* < 0.001**
	Conc. x Parasite	F_(3, 153)_ = 0.51; *P* = 0.673	F_(3, 154)_ = 5.9; ***P* < 0.001**	F_(3, 151)_ = 3.6; ***P* = 0.014**	F_(3, 154)_ = 3.4; ***P* = 0.020**
**Rate of increase**	Concentration	F_(3, 155)_ = 3.1; *P* = 0.028	F_(3, 154)_ = 11; ***P* < 0.001**	F_(3, 152)_ = 50; ***P* < 0.001**	F_(3, 154)_ = 27; ***P* < 0.001**
	Parasite presence	F_(1, 155)_ = 58; ***P* < 0.001**	F_(1, 154)_ = 2.7; *P* = 0.101	F_(1, 152)_ = 8.6; ***P* = 0.004**	F_(1, 154)_ = 5.7; ***P* = 0.018**
	Conc. x Parasite	F_(3, 155)_ = 2.2; *P* = 0.091	F_(3, 154)_ = 3.7; ***P* = 0.012**	F_(3, 152)_ = 0.42; *P* = 0.742	F_(3, 154)_ = 1.2; *P* = 0.326

For each variable, the effect of contaminant concentration, parasite treatment and their interaction (Conc. × Parasite) was evaluated. The analyses were conducted separately for each experiment (copper or tebuconazole) and for each *Daphnia* clone. Significant effects are highlighted in bold (*P* ≤ 0.025).

Neither copper nor parasite treatment had an influence on age of first reproduction (AFR), although the highest tested concentration of copper resulted in a slight delay, but only in clone 47 (Fig 1 and Table 1). Overall, copper exposure caused a slight stimulation in host reproduction (see increased fecundity and reproductive output at intermediate concentrations–Fig 1). However, the most substantial effect on *Daphnia* reproduction was caused by the parasite, which strongly reduced the host’s reproductive output in both tested clones (Fig 1 and Table 1). This effect was mainly due to parasite-induced mortality of hosts from day 14 onwards, because *Daphnia* fecundity was much less affected (compare the gap between parasite and no-parasite treatments in terms of fecundity and reproductive output–Fig 1). The per capita rate of increase confirmed the higher fitness of *Daphnia* in the no-parasite treatment. Although the effects of copper and parasite were mostly independent, a significant interaction between copper and parasite presence was found in clone 47, in terms of its reproductive output and rate of increase (Table 1). This was mostly due to a stronger decrease of these parameters in the no-parasite treatment in the highest copper concentration (see Fig 1).

### Tebuconazole x parasite

Parasite presence caused ca. 80% host mortality in the negative control (0 μg L^-1^ tebuconazole) but tebuconazole completely suppressed infection, irrespective of the nominal concentration (Fig 2). As a consequence, the main effect observed in the life history parameters was caused by contaminant concentration alone.

Tebuconazole significantly delayed AFR and decreased fecundity, reproductive output, and rate of increase, demonstrating a clear dose-response relationship independent of parasite absence/presence (Fig 2 and Table 1). The differential infection success between negative control and tebuconazole concentrations in the parasite treatment lead to a significant concentration x parasite presence interaction in *Daphnia* reproductive output (Fig 2 and Table 1). This difference was not observed for fecundity, confirming that the main effect of the parasite is on host mortality. Per capita rate of increase and reproductive output were slightly lower in the parasite treatment, compared to no-parasite treatment (Fig 2 and Table 1).

### Follow-up experiment

**Fig 3:**
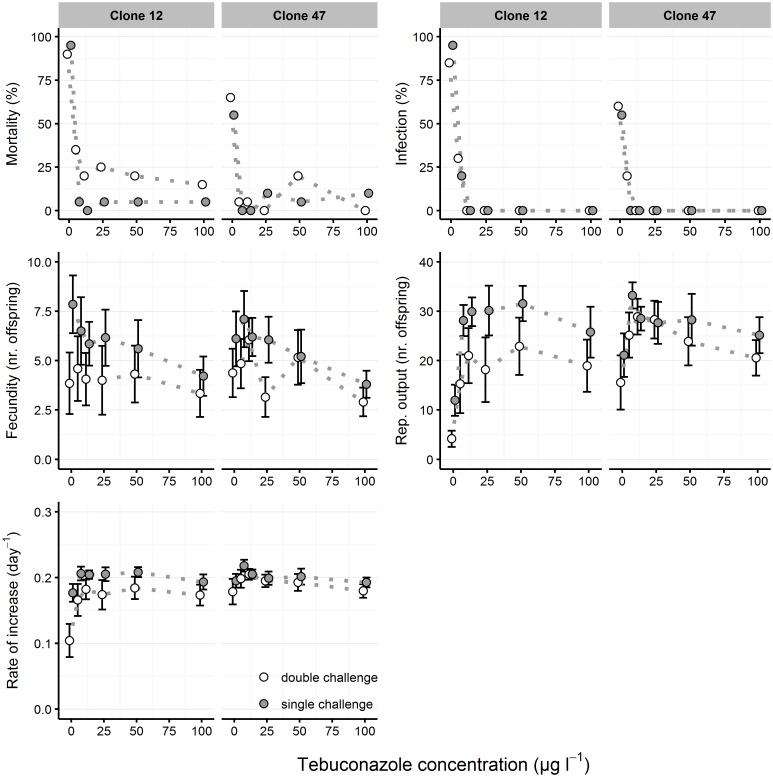
Life history parameters of *Daphnia* clones exposed to tebuconazole and parasite challenge (follow-up experiment). Two *Daphnia* clones (Clone 12 and Clone 47) were exposed to increasing tebuconazole concentrations and two levels of parasite challenge (“single challenge” vs. “double challenge”) with spores of the microparasitic yeast *M*. *bicuspidata*. Host mortality and parasite infectivity are shown as the proportion of dead or infected hosts, respectively, at the end of the experiment (day 21). Fecundity (at day 14), reproductive output (at day 21), age at first reproduction and intrinsic rate of increase are shown as mean (circle) and respective 95% confidence intervals (error bars), based on 20 experimental units per treatment. Dotted lines between data points are used to facilitate visualization of the concentration-dependent response across parasite treatments.

In the follow-up experiment, the infection procedure was also successful, although infection rates in controls (i.e. no tebuconazole) varied according to clones and levels of parasite challenge: 85–95% for clone 12 and 55–60% for clone 47 (Fig 3). This experiment demonstrated an antiparasitic effect of tebuconazole at lower concentrations than the previous ones. At 6.25 μg L^-1^ (the lowest tested concentration), tebuconazole reduced infection rates in clone 12 to 20% and 30%, and in clone 47 to 0% and 20%, for single challenge and double challenge treatments, respectively. From 12.5 μg L^-1^ onwards, tebuconazole completely suppressed infection. Overall, while the increase in the number of parasite challenges caused a slightly higher parasite success (at 0 μg L^-1^ and 6.25 μg L^-1^), this higher spore load was unable to compensate the anti-*M*. *bicuspidata* effect of tebuconazole above 12.5 μg L^-1^.

Fecundity and reproductive output were significantly affected by tebuconazole exposure but also by the level of parasite challenge (Fig 3 and Table 2). Increasing concentrations of tebuconazole significantly reduced host fecundity. However, hosts from control treatments showed reduced reproductive output and per capita rate of increase, when compared to hosts from tebuconazole treatments, due to parasite-induced mortality. This was also the pattern for per capita rate of increase (Fig 3 and Table 2). Survival, fecundity and reproductive output were consistently lower in the double challenge treatment, which was more pronounced in Clone 12. This latter finding is paradoxical, given that there is no difference in the infection success between single challenge and double challenge treatments from 12.5 μg L^-1^ onwards.

**Table 2:** Two-way ANOVA summary table of the effects of tebuconazole concentration and level of parasite challenge (follow-up experiment).

	Source	Clone 12	Clone 47
**Fecundity at day 14**	Concentration	F_(5, 221)_ = 2.8; ***P* = 0.018**	F_(5, 233)_ = 7.0; ***P* < 0.001**
	Parasite challenge	F_(1, 221)_ = 24; ***P* < 0.001**	F_(5, 233)_ = 17; ***P* < 0.001**
	Conc. x Parasite	F_(5, 221)_ = 1.1; *P* = 0.347	F_(5, 233)_ = 2.2; *P* = 0.058
**Reproductive output**	Concentration	F_(5, 238)_ = 18; ***P* < 0.001**	F_(5, 239)_ = 9.0; ***P* < 0.001**
	Parasite challenge	F_(1, 238)_ = 54; ***P* < 0.001**	F_(1, 239)_ = 9.8; ***P* = 0.002**
	Conc. x Parasite	F_(5, 238)_ = 0.56; *P* = 0.727	F_(5, 239)_ = 1.5; *P* = 0.189
**Rate of increase**	Concentration	F_(5, 239)_ = 14; ***P* < 0.001**	F_(5, 238)_ = 5.6; ***P* < 0.001**
	Parasite challenge	F_(5, 239)_ = 62; ***P* < 0.001**	F_(1, 238)_ = 11; ***P* < 0.001**
	Conc. x Parasite	F_(5, 239)_ = 3.3; ***P* = 0.007**	F_(5, 238)_ = 0.96; *P* = 0.446

For each variable, the effect of tebuconazole concentration, parasite challenge and their interaction (Conc. × Parasite) was evaluated. The analyses were conducted separately for each *Daphnia* clone. Significant effects are highlighted in bold (*P* ≤ 0.025).

## Discussion

Disease and pollution are two important stressors affecting natural populations. Interaction between these stressors can exacerbate or mitigate their otherwise independent effects [29]. This study was motivated by the need for a clarification on how pesticide pollution affects the eventual spread of diseases [23,42]. Our major finding was that two common agrochemicals (fungicides) cause different outcomes in a host-parasite (*Daphnia*-*M*. *bicuspidata*) relationship, confirming that effects of pollution on host-parasite relationships depend on the particular stressor [22,29]. Particularly, infection by *M*. *bicuspidata* was independent of copper exposure, but was suppressed in the presence of the fungicide tebuconazole.

Tebuconazole and copper are commonly used in agriculture to fight fungal pests, particularly in vineyards, and are frequently detected in superficial and runoff waters [12,31]. Despite the fact that detected concentrations for both agricultural fungicides are usually low (below 10 μg L^-1^), higher levels have been recorded in extreme runoff events (up to 263 μg L^-1^ for copper and up to 200 μg L^-1^ for tebuconazole) [13,16]. At these concentrations, tebuconazole and copper were overall noxious to *Daphnia*, decreasing survival (especially copper), reproductive output and per capita rate of increase ([3] and this study). Regarding copper, *Daphnia* reproduction was only affected at the highest tested concentration (33.06 μg L^-1^) where organisms also started to show increasing mortality. This may be due to the fact that copper, unlike tebuconazole, is an essential element for physiological processes in low doses, above which it becomes toxic [15].

Successful infection by *M*. *bicuspidata* depends on needle-like ascospores entering the host (via filtration) and piercing the gut wall [33]; thus, the more spores enter the host, the more likely they will germinate in the host´s haemolymph and develop infection. In our study, the infection procedure was successful, given that infection was always high in the negative controls (i.e. without fungicide). Infected *Daphnia* were killed within 14 to 17 days, which agrees with the described timing for development of *M*. *bicuspidata* infection [33]. Before being killed by the parasite, the fecundity of infected *Daphnia* was only slightly reduced, also in agreement with previous studies [43]. For this reason, reproductive output was more informative than fecundity, reflecting the integration of reproduction and parasite-induced mortality.

Levels of parasite challenge were determinants of host fitness: the more severe exposure (double challenge) generally led to higher infection rates than single challenge exposure, confirming the findings of Ebert et al. [44]. Also, *Daphnia* from the double challenge treatment suffered an overall stronger reduction in reproductive parameters and higher levels of mortality (Fig 3). This cannot simply be explained by a direct relationship between spore load and the strength of infection [44,45], because no visible symptoms of infection were observed under tebuconazole exposure. Possibly, mechanical damage caused by *M*. *bicuspidata* ascospores piercing the host´s gut wall [33] could explain the difference between the two levels of parasite challenge [44], even if infection was not successful.

When *Daphnia* were simultaneously exposed to copper and *M*. *bicuspidata* spores, infection was the major cause of negative effects on host reproduction due to early host death; this pattern was visible at all copper concentrations and in the negative control, confirming the independent action of both stressors. This means that copper is not toxic to the yeast; however, higher copper concentrations kill the host [3]. Unlike tebuconazole (see below), copper does not have a specific cellular target in fungal cells, which may explain its higher toxicity to the host, since *Daphnia* are particularly sensitive to metals [32]. Our results seem to contradict those of Civitello et al. [27], who demonstrated that sublethal levels of copper increased filtration rates in *Daphnia dentifera*, hence boosting *M*. *bicuspidata* spore consumption and, thus, parasite transmission. However, increased filtration rates may be less important for parasite transmission under high parasite load, such as in our case– 700 spores mL^-1^ in our study vs. 25–75 spores mL^-1^ in Civitello et al. [27]. Also, in this previous study higher copper concentrations were tested (i.e. 50–200 μg L^-1^ in contrast to 25–33 μg L^-1^ in the present experiment), which could be partially explained by their choice of a synthetic medium with chelating properties (see [32]). This stimulatory effect of copper that indirectly benefited the parasite [27] was not observed in our study.

Under tebuconazole exposure, *M*. *bicuspidata* infection was completely suppressed above 12.5 μg L^-1^. Unlike copper, the parasite was more sensitive to tebuconazole than the host. Tebuconazole, along with other azoles, is considered a fungistatic agent capable of impairing growth and preventing sporulation, particularly in yeast [46]; including species from the genus *Metschnikowia* [47]. Agricultural azole fungicides (e.g., propiconazole and tebuconazole), along with common pharmaceuticals to treat yeast infections (e.g., clotrimazole and fluconazole), have been detected in the environment, raising concerns about putative negative effects on non-target organisms [17,48] and in important microbe-mediated ecological processes [13,47]. In our case, increasing concentrations of tebuconazole significantly affected reproductive and demographic parameters in *Daphnia*, but the most striking result was that infection signs were suppressed at environmentally relevant concentrations (i.e. from 6.25 μg L^-1^ up). In fact, although tebuconazole concentrations up to 200 μg L^-1^ have been detected in the field (reviewed by Zubrod et al. [13,16]), typical concentrations in the aquatic environment are below 10 μg L^-1^ [12,31,49]. Thus, even low, environmentally-relevant concentrations of tebuconazole can cause the termination of parasite epidemics, with unknown ecological consequences.

Although there were no signs of infection in organisms exposed to tebuconazole, daphniids displayed slightly lower fitness under parasite challenge (see reproductive output data–Fig 2). It might be that *M*. *bicuspidata* growing inside the host’s body cavity could be causing injury or using *Daphnia*’s vital resources. Further research is needed to clarify whether this reduction in host fitness results from mechanical injury by spores piercing the gut wall (discussed above) or from the yeast’s parasitic action. Azole fungicides lead to a depletion in ergosterol necessary to membrane functioning, thus inhibiting fungal growth and eventually causing their death [50,51]. Possible fungicidal or fungistatic effects of tebuconazole could occur at multiple stages: a) suppression of initial spore germination (after being filtered by *Daphnia*); b) growth inhibition due to depletion of ergosterol; c) death of yeast cells that were able to germinate from initial spores; d) reproduction (i.e., sporulation) impairment. It is not clear whether these effects are reversible or not, as *M*. *bicuspidata* may have remained latent in the *Daphnia* body cavity during tebuconazole exposure, thus delaying the infection instead of suppressing it. This delay scenario could not be confirmed in our study due to the short duration of the assay, and further experiments need to be conducted to address that issue.

Antagonistic effects between fungicides and parasitism have been reported previously. Blanar et al. [28] showed that exposure to zinc and copper reduced survival and fecundity of a juvenile Atlantic salmon ectoparasite without affecting the host; Hanlon et al. [52] demonstrated that exposure of frog tadpoles to the fungicide thiophanate-methyl and a chytrid fungus resulted in a clear suppression of the infection. Such phenomena may be widespread, and should not be underestimated. Many antifungal formulations present in agrochemicals, pharmaceuticals and personal care products may affect non-target fungal parasites, which otherwise control natural populations of a variety of hosts, including cyanobacteria [53,54], protists [54,55], plants [56], invertebrates [57], fishes [58] and amphibians [52,59]. Also, parasites in general interfere with energy flow and are important drivers of genetic diversity [18]. Thus, changes in the outcome of host-parasite relationships may upscale to population, community and ecosystem level [23,45,60]. While the suppression of infection (as detected in the tebuconazole treatment) may seem positive from the host´s point of view, the exact consequences of an antagonism between pollution and disease, as shown here, are still poorly understood. The growing needs of the human population and the challenges of climate change will increase the use of pesticides (including fungicides), which will likely cause deleterious effects in non-target fungi and bacteria, such as microparasites and decomposers. It is of primordial importance that such phenomena are not overlooked, and that protective measures are implemented to preserve important and diverse ecological processes that are mediated by these microbial communities.

## Supporting information

S1 Table Nominal and analytical concentrations (mean ± SD, n = 2) for copper (free ion, Cu^2+^) and tebuconazole. (DOCX)

S1 Fig Stages of *Metschnikowia bicuspidata* infection in *Daphnia galeata × longispina*. (A) healthy, uninfected female; (B) early development of infection signs–body becomes more opaque with the presence of ascospores (not all of them mature yet); (C) late development of infection signs–body cavity is filled with needle-like ascospores; (D) detail of ascospores released by dead hosts.
(DOCX)
