# Supplementary material for: Interplay between fungicides and parasites: Tebuconazole, but not copper, suppresses infection in a Daphnia-Metschnikowia experimental model
Source: PLoS One. 2017 Feb 23;12(2):e0172589. doi: 10.1371/journal.pone.0172589 (PMC5322920; doi:10.1371/journal.pone.0172589)
Supplement: S1 Table — (DOCX) [file pone.0172589.s001.docx]

**S1 Table –** Nominal and analytical concentrations (mean ± SD, n=2) for copper (free ion, Cu^2+^) and tebuconazole.

|  | Nominal concentrations (µg l^-1^) | Measured concentrations (µg l^-1^) | % deviation from nominal concentrations |
| --- | --- | --- | --- |
| Copper experiment | 25.0 | 27.5 ± 3.5 | 10% |
|  | 33.1 | 38.5 ± 0.7 | 16% |
| Tebuconazole experiment | 153.6 | 155.0 ± 7.1 | 0.9% |
|  | 240.0 | 255.0 ± 7.1 | 6.3% |
| Follow-up experiment | 6.25 | 7.35 ± 0.21 | 17.6% |
| (tebuconazole) | 100 | 110 ± 0.0 | 10% |
