# Supplementary material for: Interplay between fungicides and parasites: Tebuconazole, but not copper, suppresses infection in a Daphnia-Metschnikowia experimental model
Source: PLoS One. 2017 Feb 23;12(2):e0172589. doi: 10.1371/journal.pone.0172589 (PMC5322920; doi:10.1371/journal.pone.0172589)
Supplement: S1 Fig — (A) healthy, uninfected female; (B) early development of infection signs–body becomes more opaque with the presence of ascospores (not all of them mature yet); (C) late development of infection signs–body cavity is filled with needle-like ascospores; (D) detail of ascospores released by dead hosts. (DOCX) [file pone.0172589.s002.docx]

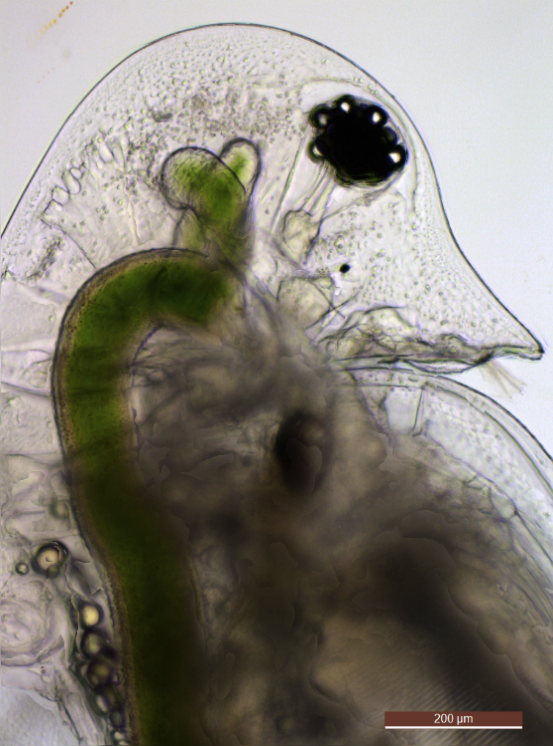

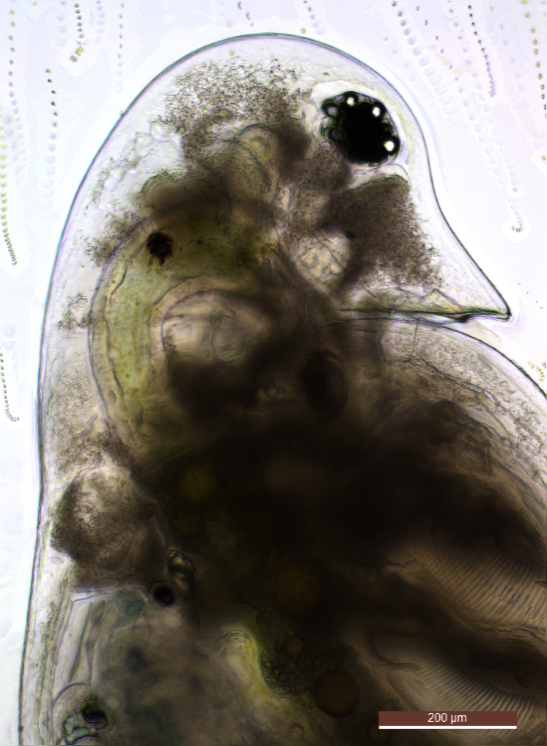

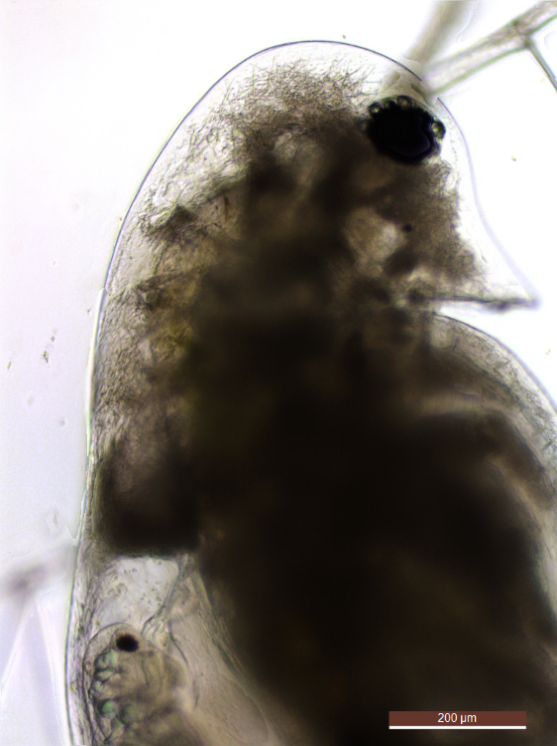

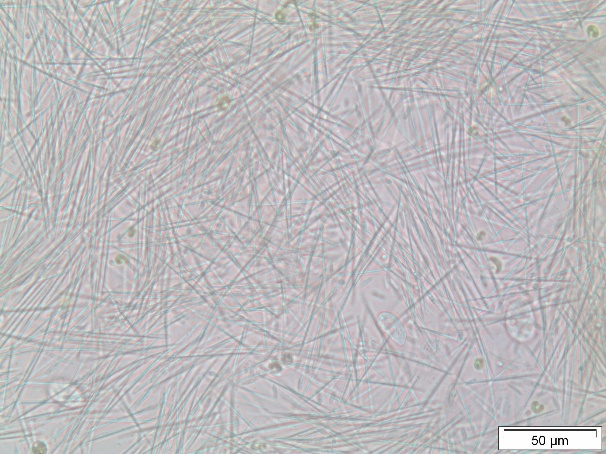


D

C

B

A

**S1 Fig. Stages of *Metschnikowia bicuspidata* infection in *Daphnia galeata × longispina*.**

(A) healthy, uninfected female; (B) early development of infection signs – body becomes more opaque with the presence of ascospores (not all of them mature yet); (C) late development of infection signs – body cavity is filled with needle-like ascospores; (D) detail of ascospores released by dead hosts.
